# Supplementary material for: DMRforPairs: identifying Differentially Methylated Regions between unique samples using array based methylation profiles
Source: BMC Bioinformatics. 2014 May 15;15:141. doi: 10.1186/1471-2105-15-141 (PMC4046028; doi:10.1186/1471-2105-15-141)
Supplement: Additional file 2 — DMRforPairs output for the comparison of A431-MCF7 and NA17018-NA17105. Please start from the HTML files in each folder. Available via the BMC Bioinformatics website. [file 1471-2105-15-141-S2.zip › 1394847754114233_MOESM2_ESM/A431_MCF7/figures/37.pdf]

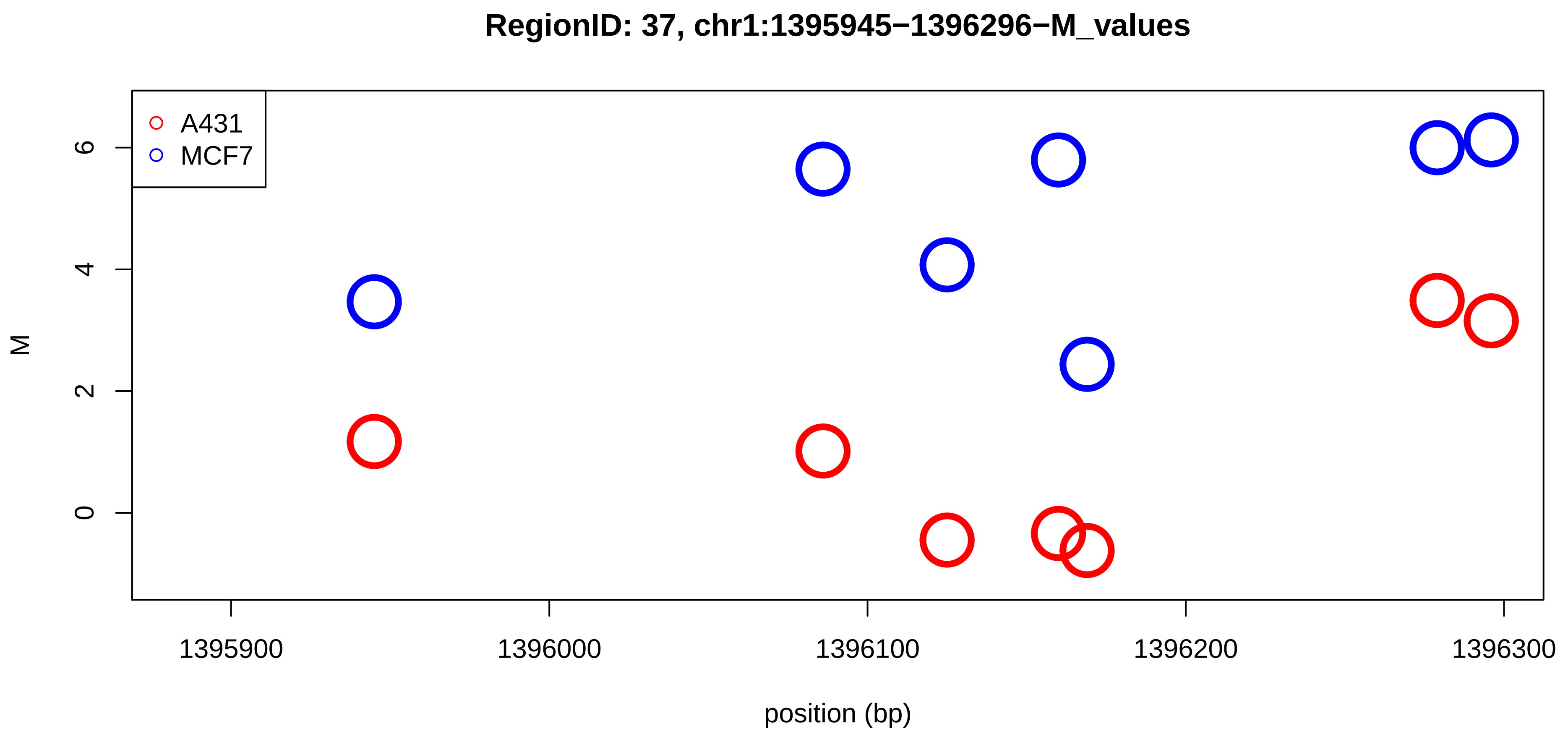

RegionID: 37, chr1:1395945−1396296−M_values
A431
MCF7
M
0
2
4
6
1395900
1396000
1396100
1396200
1396300
position (bp)

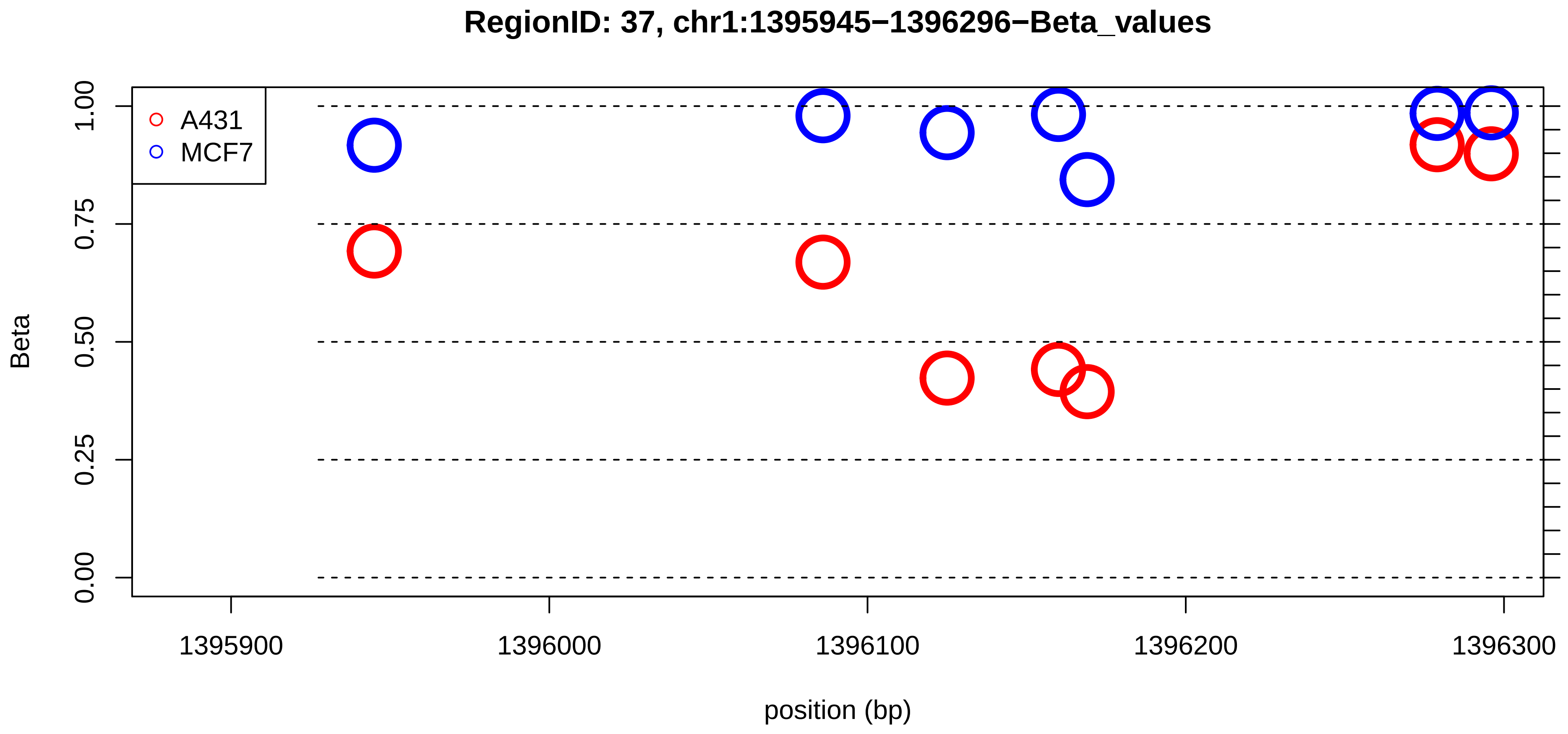

RegionID: 37, chr1:1395945−1396296−Beta_values
A431
MCF7
Beta
0.00
0.25
0.50
0.75
1.00
1395900
1396000
1396100
1396200
1396300
position (bp)

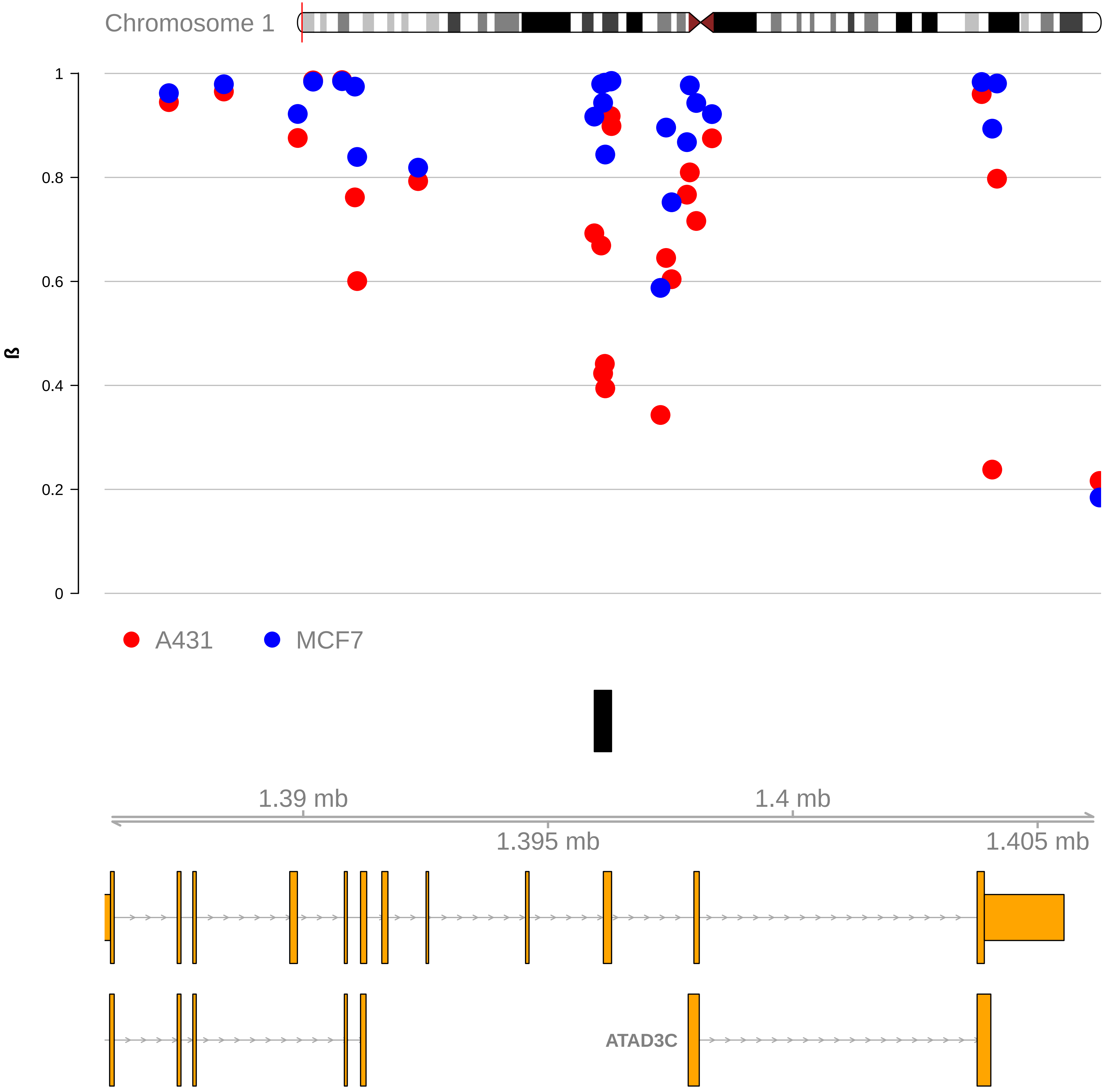

Chromosome 1
1
0.8
0.6
0.4
0.2
0
ß
A431
MCF7
1.39 mb
1.4 mb
1.395 mb
1.405 mb
ATAD3C
